# DoBo: Protein domain boundary prediction by integrating evolutionary signals and machine learning

**DOI:** 10.1186/1471-2105-12-43

**Published:** 2011-02-01

**Authors:** Jesse Eickholt, Xin Deng, Jianlin Cheng

**Affiliations:** 1 Department of Computer Science, University of Missouri, Columbia, MO 65211, USA; 2 Informatics Institute, University of Missouri, Columbia, MO 65211, USA; 3 C. Bond Life Science Center, University of Missouri, Columbia, MO 65211, USA

## Abstract

**Background:**

Accurate identification of protein domain boundaries is useful for protein structure determination and prediction. However, predicting protein domain boundaries from a sequence is still very challenging and largely unsolved.

**Results:**

We developed a new method to integrate the classification power of machine learning with evolutionary signals embedded in protein families in order to improve protein domain boundary prediction. The method first extracts putative domain boundary signals from a multiple sequence alignment between a query sequence and its homologs. The putative sites are then classified and scored by support vector machines in conjunction with input features such as sequence profiles, secondary structures, solvent accessibilities around the sites and their positions. The method was evaluated on a domain benchmark by 10-fold cross-validation and 60% of true domain boundaries can be recalled at a precision of 60%. The trade-off between the precision and recall can be adjusted according to specific needs by using different decision thresholds on the domain boundary scores assigned by the support vector machines.

**Conclusions:**

The good prediction accuracy and the flexibility of selecting domain boundary sites at different precision and recall values make our method a useful tool for protein structure determination and modelling. The method is available at http://sysbio.rnet.missouri.edu/dobo/.

## Background

It has been well over thirty years since Wetlaufer formally introduced what he termed structural regions of a protein chain. Such regions were portions of the peptide sequence which assumed a compact structure [1]. In modern parlance, these units are known as domains. Protein domains are structural, functional and evolutionary units and are the building blocks of larger proteins [2]. In recent years, the identification and delineation of protein domains has become more prominent as this information eases the determination of protein structure by experimental means and can also speed up computational approaches for protein structure prediction [3,4].

Due to the large amounts of data being generated by today's technology, human experts can no longer keep up. It is simply not possible to visually identify and annotate such a large number of domains. Thus, computational approaches are needed to fill the gap.

At present, computational methods for protein domain prediction can be roughly dichotomized as either template-based or *ab-initio*. Most template-based approaches attempt to find homologous sequences in one of the many existing domain databases and then infer from these sequences the domain(s) of the protein in question. Of course the drawback to this approach is that it will only work if a domain is conserved and has already been deposited in a database. A few template based methods [5,6] take a different approach and build a 3D model using structural templates found by fold recognition. The domains are then derived from the generated model. *Ab-initio *methods make predictions based solely on the primary sequence of a protein and therefore work regardless of the novelty of the protein at hand. Traditional methods for this type of approach include sequence comparison, neural networks and statistical analysis [7-13]. Some of the newer *ab-initio *approaches construct an ensemble of 3D models via *de novo *modelling techniques which are then analyzed and parsed for domain boundaries[5,14]. Finally, there do exist a small number of hybrid methods which combine both template based and *ab-initio *approaches into one comprehensive package [15,16].

For proteins without homology to known structures, *ab-initio *approaches are the only choice. Unfortunately, the accuracy of their domain boundary predictions is still too low for general, practical use [11,12,17,18]. Most *ab-initio *methods can be classified into two sub-categories: comparative sequence analysis [7,8,19-24] and direct boundary prediction [12,17,25]. Most comparative sequence analysis methods use pairwise sequence alignment similarity to cluster sequence segments into domains [7,8,19-24]. The direct boundary prediction methods try to identify domain boundary regions such as domain linkers, exploiting their sequence and structural biases [26-28]. This is done using machine learning techniques [11,12,17] which are trained on known domain boundaries extracted from domain classification databases such as CATH [29], SCOP [30] and DALI [31,32]. Still, because these methods need to scan several hundred positions (i.e. to cover the length of the protein) and rely on inputs containing very weak domain boundary information, they often suffer from low accuracy.

Here we present DoBo, a new *ab-initio *method we have developed to exploit evolutionary domain boundary signals embedded in homologous proteins. This reduces the search space of domain boundaries and in turn improves domain boundary prediction. It is well known that during evolution genes may undergo recombination to produce complex domain architectures via gene fusion [33], gene fission [33,34], domain duplication and domain swapping [34-38]. Thus evolutionary related domains may exist in different forms in different organisms [39]. Some exist as a component of multi-domain proteins and some as standalone single domain proteins [40,41]. When a multi-domain protein sequence is searched against a protein sequence database (e.g. NCBI non-redundant sequence database [42]), proteins containing domains similar to the target protein are returned which often reveal the domain architecture of the target protein. We integrate evolutionary domain boundary signals with machine learning classification into a two-step prediction procedure. First, we leverage evolutionary information and generate domain boundary signals which identify potential domain boundary sites. These sites are then further examined and classified as boundary or non-boundary sites using machine learning methods.

## Methods

### Data Set Preparation

The starting point for our dataset was a collection of proteins curated for the DOMpro package [11]. From this set, we extracted only those proteins whose domain number agreed in both SCOP (v 1.75) and CATH (v 3.3.0) [29,30]. Then we removed any protein whose length was less than 90 residues long as these sequences were incapable of generating signals. This resulted in a final data set containing a total of 628 protein sequences, 186 of which were multi-domain proteins and 442 were single domain proteins. The domain definitions used for domain boundary signal classification for training and evaluation are those provided by CATH. The PDB identifiers and domain definitions for these proteins can be found online [43].

### Identification and Classification of Domain Boundary Signals

**Figure 1:**
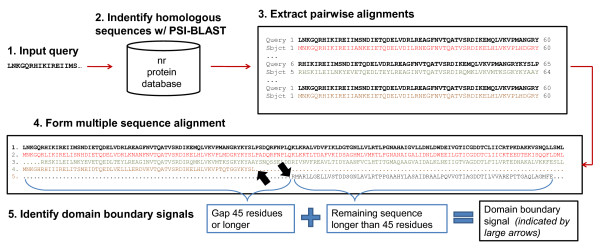
**Procedure to identify and extract domain boundary signals**. To identify domain boundary signals for a target, homologous sequences are found using PSI-BLAST. The pairwise alignments generated by PSI-BLAST are used to form a multiple sequence alignment with the query sequence as the anchor. A domain boundary signal is defined as a gap which begins at the N or C terminal end of a sequence in the msa and extends continuously for at least 45 residues. With the gaps removed the remaining sequence must be at least 45 residues long for a signal to be generated. Here we see two domain boundary signals for 1B4A (location indicated by large arrows).

To detect putative domain boundary signals for a protein, PSI-BLAST [44] is used to generate a multiple sequence alignment (msa). This is achieved by running PSI-BLAST to search a query sequence against the NCBI non-redundant protein sequence database [42] (i.e. nr-database) for 3 iterations with an e-value of .001. Then the pairwise alignments generated by PSI-BLAST are extracted and used to form a multiple sequence alignment anchored on the query sequence. A domain boundary signal is defined as a gap which begins at the N or C terminal end of a sequence in the msa and extends continuously for at least 45 residues. We make an additional stipulation that with the gaps removed the remaining sequence must be at least 45 residues long for a signal to be generated. The location of the domain boundary signal is defined to be the first non-gap residue in the sequence. Figure 1 illustrates this process and shows two domain boundary signals for protein 1B4A.

When extracting domain boundary signals from a multiple sequence alignment, each sequence from the msa is processed in order of increasing PSI-BLAST e-value. The location of each domain boundary signal is noted and aggregated to a list of all the signals for the protein.

The collection of domain boundary signals stops when all of the sequences in the msa have been processed or whenever signals have been generated at 35 unique residue locations, whichever comes first. It is worth noting that these parameter values used to generate putative domain boundary sites are adjustable and may have some impact on the sensitivity and specificity of domain boundary prediction.

Domain boundary signals are classified as one of three possible types: false boundary, near boundary, or away boundary. False boundary signals are those generated from a single domain protein. Near boundary signals are those which occur within 20 residues of any domain boundary in a multi-domain protein. The remaining signals come from multi-domain proteins and correspond to away boundary signals as they take place more than 20 residues away from a true domain boundary. The 20-residue threshold is in accordance with previous research [11,17,45].

### Machine Learning Prediction Protocol

To predict domain boundaries, each domain boundary signal was classified using a support vector machine (SVM) [46]. As support vector machines are binary classifiers, we perform the classification using two separate support vector machines in a two stage process. The first SVM (Task-1) was trained to separate false boundary signals from near and away boundary signals (i.e. to discriminate signals generated from a single domain protein from those generated from a multi-domain protein). The second SVM (Task-2) was trained solely on signals from multi-domain proteins and was charged with discriminating near boundary signals from away boundary signals.

To determine if a protein is single domain or multi-domain, we first classify all domain boundary signals as false signals or near/away signals. If a protein has one or more near/away signals, it is classified as a multi-domain protein. Those proteins which only generate signals classified as false signals or do not generate any signal at all are classified as single domain proteins. Domain boundaries are predicted based directly on the output of SVMlight. For each domain boundary signal, a set of features is fed into SVMlight and output is generated. Generally speaking, for Task-2 if the output is positive, i.e. greater than 0, then a domain boundary is predicted at that signal site. It is also possible to set a different decision threshold and determine predicted domain boundaries with respect to that new threshold.

### Sequence Encoding and Training Method

Both Task-1 and Task-2 SVM predictors were trained using the SVM light package [47]. The features used in training came from a window of 41 residues centered around the signal site. For each residue in the window, 21 features were used for a sequence profile (i.e. normalized frequencies of 20 residues plus a gap) and 5 features (i.e. helix, strand, loop, buried, exposed) encoded the secondary structure and solvent accessibility as predicted by the SSpro suite [48]. In addition to these residue specific features, we also added 3 signal specific features such as the position of the signal with respect to the N terminal (residue index divided by 100), position with respect to the C terminal (protein length minus residue index divided by 100) and a count of boundary signal sites within 5 residues. Additionally, as a protein specific feature we used the length of the sequence divided by 100. The final feature was a measurement of the total number of signals generated by all of the sequences in the msa within a 5 residue neighbourhood of the signal site. This local sum was calculated for each residue in the sequence and then converted to z-scores. The z-score for the signal site was added as the final feature and this resulted in a feature vector containing a total of 1071 features.

For both Task-1 and Task-2 SVM predictors, we used a radial basis kernel function and set gamma to "0.015" according to a leaving one out cross validation (LOOCV) procedure. For the purposes of training and evaluation we performed 10 fold cross validation, splitting the proteins up into 10 set of approximately equal size. For Task-1 we used all proteins in our dataset while for Task-2 we limited ourselves to those targets known to be multi-domain proteins.

## Results

### Signal Coverage of Domain Boundaries

**Figure 2:**
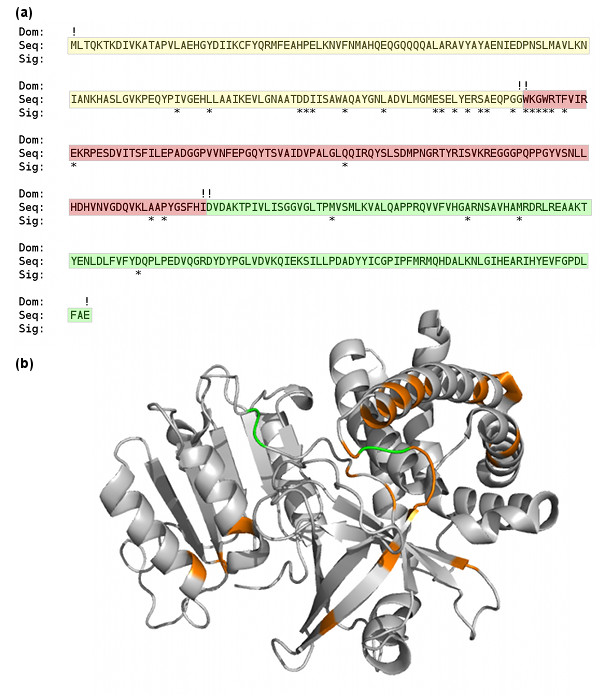
**Domain boundary signal sites for 1CQX**. (a) Domain boundary signal site locations which were extracted from a multiple sequence alignment for chain A of protein 1CQX. Signals (denoted by '*") were generated at 28 different residues across this three domain protein. The true domains and domain boundaries are also indicated (boundaries with an '!'). Note that all domain boundaries have signals nearby indicating good coverage of the domain boundaries. (b) Structural plot for chain A of protein 1CQX. The locations of domain boundary signals are shown in orange and true domain boundaries are green.

To ascertain the usefulness of domain boundary signals generated by multiple sequence alignments, we calculated the percentage of domain boundaries which had a signal within 20 residues. When calculating this value, we excluded the domain boundary closest to each terminal end of the protein sequence (i.e, the first and last domain boundaries with respect to the residue index were not considered). For our dataset, there were 462 such boundaries and we found that 391 had a domain boundary signal within 20 residues. Thus, 84.6% of domain boundaries had a signal nearby. Figure 2 illustrates the distribution of the domain boundary signals generated for 1CQX along with the true domain boundaries.

### Site Level Evaluation of Domain Boundary Signals

**Figure 3:**
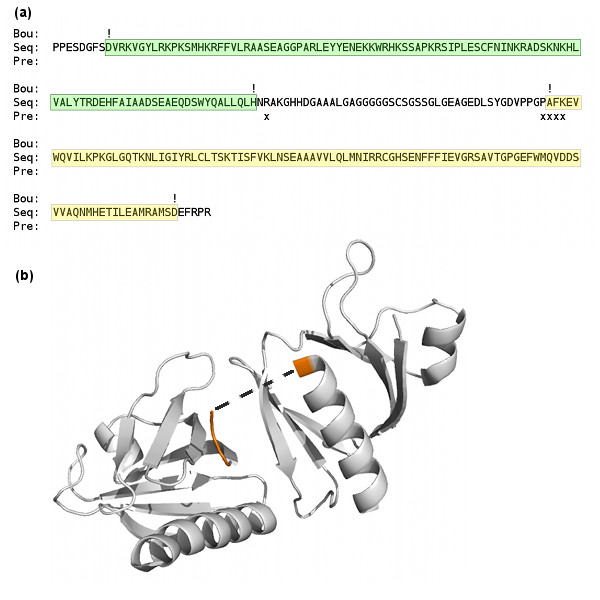
**Domain boundary predictions for 1QQG**. (a) True domains and domain boundaries (boundaries indicated by '!') and the predicted domain boundaries (indicated by 'x') for chain A of protein 1QQG, a two domain protein with a domain linker delineated by "!". Both domain boundaries are accurately predicted. These predictions were made using a decision threshold of 0.5 (b) Structural plot for chain A of protein 1QQG. The predicted domain boundaries are shaded orange. The linker between the two domains could not be structurally determined (i.e., its coordinates were not available) and is therefore represented by the dashed line.

Table 1 reports the results at site level for the two binary classification tasks: Task 1, near/away boundary signals (positive) VS false boundary signals (negative) and Task 2, near boundary signals (positive) VS away boundary signals (negative). For site level evaluation for Task 1, overall classification accuracy (i.e., percent of correct predictions) is 80% using 10-fold cross validation on all the proteins in the data set. The overall classification accuracy for Task 2 predictions was 74% using 10-fold cross validation. Using leaving one out cross validation procedure (LOOCV), the accuracy is slightly higher (i.e. 81% for Task 1 and 76% for Task2). Figure 3 shows one example where domain boundaries were correctly predicted.

**Table 1:** Boundary site signal classification results for Task-1 and Task-2 using both 10-fold cross validation and leaving one out cross validation.

Classification Task	Overall Acc. Using 10-Fold Cross Validation	Overall Accuracy Using LOOCV	
Task 1 (near/away boundary VS false boundary)	.80	.81	

Task 2 (away boundary VS near boundary)	.74	.76	

**Figure 4:**
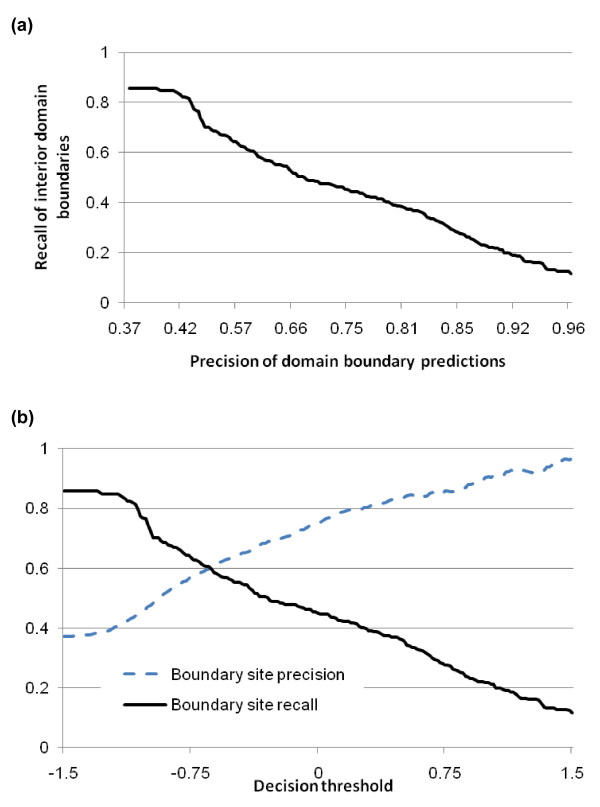
**Domain boundary prediction results on multi-domain proteins**. (a) We calculated the precision of domain boundary predictions and recall of true domain boundaries at varying decision thresholds. The recall value is calculated for domain boundaries which occur at least 40 residues from the N or C terminal end of a sequence. A domain boundary prediction is considered correct if it occurs within 20 residues of a true domain boundary. (b) Plot of precision and recall with respect to the decision threshold. The break-even point (precision = recall) is 60%.

One key application of domain boundary prediction is to select positions to cut a large protein into foldable units for structure determination or prediction. In order to facilitate this application, we study how the precision and recall of domain boundary predictions change according to decision thresholds on domain boundary scores predicted by the support vector machines. Figure 4 illustrates a plot of the precision and recall for domain boundary sites as a function of the decision threshold based on 10-fold cross validation. The decision threshold was the value used in conjunction with the output of SVMlight to discriminate between near and away boundary sites. It was varied from -1.5 to 1.5 and at each threshold, signals were classified and the precision and recall were calculated for the sites classified as near boundary. The break-even point (i.e. precision = recall) was found to be 60%, which means 60% of true domain boundaries can be predicted at a precision of 60%. We believe domain boundary predictions at this accuracy level can used to effectively inform protein structure determination and modelling. For the purposes of these calculations, any signal classified as near boundary and was within 20 residues of a true domain boundary was counted as a correct prediction. For recall, we calculated the percentage of true domain boundaries which were more than 40 residues way from the N or C terminal and had a near boundary signal with in 20 residues.

### Protein Level Results

Table 2 reports the classification accuracy of our prediction protocol when classifying a protein as single or multi domain based on 10-fold cross validation. We considered a protein to have multiple domains if it generated at least one domain boundary signal which was classified as a near/away signal by the Task-1 classifier. Overall 515 of the 628, or 82% of the proteins considered, were correctly classified as either a single or multi domain protein. The precision and recall for classifying a protein as single domain were 0.88 and 0.86, respectively. For multi domain proteins, the performance was slightly less with the precision being 0.68 and the recall 0.72.

**Table 2:** Classification of proteins as single or multi-domain

Overall Acc.	Single Dom. Precision	Single Dom. Recall	Multi-Dom. Precision	Multi-Dom Recall
0.82	0.88	0.86	0.68	0.72

Using the results from Task 1, we classified proteins as a single or multi-domain. Any protein which generated at least one boundary signal which was classified as a near/away boundary signal was considered a multi-domain protein.

### Comparison with Other Domain Boundary Predictors on CASP9 Targets

As an additional assessment of our method, we evaluated its performance along with that of two additional *ab-initio *domain boundary predictors on the targets from the Critical Assessment of Techniques for Protein Structure Prediction (CASP9). The additional predictors considered were DOMPro and PPRODO [11,13]. The sequences and domain definitions for these targets were obtained directly from the CASP9 server [49]. To evaluate the performance of the predictors at the protein level (i.e. single or multi-domain classification), all CASP9 targets with domain definitions were used. When evaluating domain boundary predictions, we limited the dataset to 14 multi-domain targets which had continuous domain definitions as these methods were largely designed to handle domains without non-continuous segments. The results of this evaluation are summarized in Tables 3 and 4. Table 5 lists the multi-domain targets used and their corresponding domain definitions.

**Table 3:** Classifcation of CASP9 targets as single or multi-domain

Predictor	Accuracy	Single Dom. Precision	Single Dom. Recall	Multi-Dom. Precision	Multi-Dom Recall
DOMPro	0.72	0.82	0.84	0.30	0.28

PPRODO	0.63	0.84	0.65	0.30	0.56

DoBo	0.78	0.90	0.81	0.50	0.68

Using DOMPro, PPRODO and our method DoBo, we classified all CASP9 targets as single or multi-domain. For PPRODO, predictions were based on the authors' documented procedure for predicting domain number [13]. For Dobo, any target which generated at least one boundary signal which was classified as a near/away boundary signal was considered to be multi-domain.

**Table 4:** Precision and recall of domain boundary predictions on CASP9 continuous, multi-domain targets

Predictor	Precision of Domain Boundary Prediction	Recall of Domain Boundaries
DOMPro	0.50	0.14

PPRODO	0.50	0.52

DoBo	0.49	0.70

For the 14 continuous, multi-domain targets from CASP9, we used DOMPro, PPRODO and our method DoBo to predicted domain boundaries. Only domain boundary predictions which were more than 40 residues from the N or C terminal end of a sequence were considered. A domain boundary prediction is considered correct if it occurs within 20 residues of a true domain boundary. The recall value is calculated for domain boundaries which occur at least 40 residues from the N or C terminal end of a sequence.

**Table 5:** Continuous, multi-domain CASP9 targets and domain definitions

Target	Domain Definitions
T0529	7-339, 364-561

T0537	65-350, 351-381

T0542	2-302, 303-585 *

T0548	12-46, 47-106

T0550	31-117, 178-339

T0553	3-65, 66-136

T0571	32-196, 197-331

T0575	1-63, 64-216 *

T0582	2-122, 123-221

T0586	5-84, 85-123

T0596	6-58, 59-188

T0600	17-75, 76-122

T0608	29-117, 118-278

T0611	3-55, 56-213

The target numbers and domain definitions used when evaluating domain boundary predictions on the CASP9 dataset. For targets T0542 and T0575, a portion of the domain definition was disjoined. These disjoined portions were consolidated into one range.

## Discussion

One immediate benefit of this new domain boundary prediction process is the combination of the strengths of machine learning and evolutionary signals. Evolutionary signals embedded in multiple sequence alignments help significantly reduce the search space. As mentioned, the domain boundary signal embedded in the primary sequence is very weak. Any reduction in the search space which does not eliminate domain boundary sites will likely increase overall accuracy of domain boundary prediction as it will reduce the chance of false positives. For our dataset, the average sequence length is 210 residues while the average number of domain boundary signals generated per protein is 23. This is a significant reduction in the number of sites that must be classified. Remarkably, this 10-fold reduction in search space does not severely hamper the search for domain boundaries as the number of domain boundaries which have a signal nearby is still quite high, at slightly under 85%.

We have also demonstrated that not only are signals generated near domain boundary sites, but they also contain useful information which can be used to classify them. The machine learning method, which incorporates sequence profiles, secondary structures, relative solvent accessibilities and positional information of putative boundary sites, can produce scores to rank, select and classify the largely reduced set of putative domain boundary sites. Our two-tiered classification approach allows proteins to be classified as single or multi-domain and the boundary signals in multi-domain proteins can be further processed in a task specific way. When classifying signals as near or away boundary signals, our method allows the user to specify a threshold to meet his or her needs. The threshold can be decreased to boost recall or it can be raised to better precision. This is a stark contrast to many other methods which fix the threshold and do not allow for application specific use.

Figure 4(b) shows the effects of varying the decision threshold on both precision and recall for domain boundaries. This figure demonstrates the performance of our approach on the domain boundary site level as no distinction is made as to where the sites are located. In an attempt to gage performance on the protein level, we varied the decision threshold and calculated the precision and recall of domain boundary predictions for only those proteins that contained domain boundary predictions. Using a threshold of "0", we found that our method made a domain boundary prediction for 137 of the 186 multi-domain proteins (roughly 74%). When we evaluated the precision and recall of domain boundary predictions on only those 137 proteins we found those values to be .75 and .68 respectively. This further illustrates the usefulness of the decision threshold.

In addition to the decision threshold, there are a number of other parameters that can be set and modified. With respect to the signal generation process, it is possible to vary the e-value threshold of the PSI-BLAST search, the minimum signal gap, minimum domain length and unique signal site limit. Overall, we found that the method is quite robust within a range of reasonable parameter values and the tuning of these parameters usually involves some minor trade-offs between different prediction objectives. For instance, we set a shorter minimum domain and signal gap length, and used an older version of the NCBI non-redundant database when generating domain boundary signals and this yielded a slightly higher overall accuracy for Task 1 and Task 2 (i.e. ~85% and ~77% respectively using a LOOCV procedure), but a lower precision and recall at the break-even point (i.e. ~53%). The final values used for these parameters were chosen empirically based on coverage of domain boundaries by signals, the average number of signal sites per protein and the break-even point. Another parameter that can be set is the number of sequences to be considered from the multiple sequence alignment. We found that considering all sequences in a multiple sequence alignment can sometimes be detrimental to the overall performance. While it is true that allowing more sequences for consideration often increases the number of signals and hence increases the coverage of domain boundaries, it does so at the cost of enlarging the search space. We also found that number of signal generated for a protein has no direct bearing on performance. The precision and recall of domain boundary predictions for proteins generating fewer than 10 signals is comparable to that of proteins which generate many more signals.

A drawback to our approach is that by limiting the search space by means of evolutionary signals, our method is dependent on the generation of those signals. That is to say if no signals are generated then domain boundary predictions cannot be made. We have found that when signals are not generated, the most common cause is that the length of the protein is too short. For a domain boundary signal to be generated it must occur at least 45 residues from the N or C terminal and the resulting domain must be at least 45 residues long. This effectively means that proteins less than 90 residues in length are incapable of generating signals. In practice, this limitation does not pose any serious problem as such proteins are likely to be single domain and hence there are no boundaries to detect. Another reason that signals might not be generated is if a significant number of homologs cannot be identified during the PSI-BLAST search. This does occasionally happen and in this case the method will not work.

## Conclusions

We developed a two-step procedure to integrate machine learning and domain evolutionary signals to improve domain boundary prediction. The evolutionary domain signals extracted from multiple sequence alignments of query proteins and their homologs can reduce the space of the domain boundary search by about 10 fold while retaining the majority of true domain boundaries. The further application of support vector machines together with other sequence-derived features can effectively score and classify these putative boundaries in order to identify true domain boundaries. The numerical scores assigned to the predicted domain boundaries make it possible to select domain boundaries at different precision and recall values. This flexibility and the good prediction accuracy make this method a valuable tool for protein structure determination and prediction. It is available at http://sysbio.rnet.missouri.edu/dobo/.

## Authors' contributions

JC designed and implemented the first version of the method and conducted the initial experiments. JE implemented the second version of the method, added some new features, and carried out the remaining experiments. XD converted some programs from Perl into C++. JE and JC drafted the manuscript. All the authors read, edited and approved the final manuscript.
